# Thoracic dorsal root ganglion stimulation reduces acute myocardial ischemia induced ventricular arrhythmias

**DOI:** 10.3389/fnins.2023.1091230

**Published:** 2023-01-30

**Authors:** Yuki Kuwabara, Kimberly Howard-Quijano, Siamak Salavatian, Tomoki Yamaguchi, Samir Saba, Aman Mahajan

**Affiliations:** ^1^Department of Anesthesiology and Perioperative Medicine, University of Pittsburgh, Pittsburgh, PA, United States; ^2^Department of Anesthesiology and Perioperative Medicine, University of Pittsburgh Medical Center, Pittsburgh, PA, United States; ^3^Division of Cardiology, Department of Medicine, University of Pittsburgh Medical Center, Pittsburgh, PA, United States

**Keywords:** myocardial ischemia, dorsal root ganglion stimulation, autonomic nervous system, cardiac electrophysiology, ventricular arrhythmias, sudden cardiac death

## Abstract

**Background:**

Dorsal root ganglion stimulation (DRGS) may serve as a novel neuromodulation strategy to reduce cardiac sympathoexcitation and ventricular excitability.

**Objective:**

In this pre-clinical study, we investigated the effectiveness of DRGS on reducing ventricular arrhythmias and modulating cardiac sympathetic hyperactivity caused by myocardial ischemia.

**Methods:**

Twenty-three Yorkshire pigs were randomized to two groups, which was control LAD ischemia-reperfusion (CONTROL) or LAD ischemia-reperfusion + DRGS (DRGS) group. In the DRGS group (*n* = 10), high-frequency stimulation (1 kHz) at the second thoracic level (T2) was initiated 30 min before ischemia and continued throughout 1 h of ischemia and 2 h of reperfusion. Cardiac electrophysiological mapping and Ventricular Arrhythmia Score (VAS) were assessed, along with evaluation of cFos expression and apoptosis in the T2 spinal cord and DRG.

**Results:**

DRGS decreased the magnitude of activation recovery interval (ARI) shortening in the ischemic region (CONTROL: −201 ± 9.8 ms, DRGS: −170 ± 9.4 ms, *p* = 0.0373) and decreased global dispersion of repolarization (DOR) at 30 min of myocardial ischemia (CONTROL: 9546 ± 763 ms^2^, DRGS: 6491 ± 636 ms^2^, *p* = 0.0076). DRGS also decreased ventricular arrhythmias (VAS–CONTROL: 8.9 ± 1.1, DRGS: 6.3 ± 1.0, *p* = 0.038). Immunohistochemistry studies showed that DRGS decreased % cFos with NeuN expression in the T2 spinal cord (*p* = 0.048) and the number of apoptotic cells in the DRG (*p* = 0.0084).

**Conclusion:**

DRGS reduced the burden of myocardial ischemia-induced cardiac sympathoexcitation and has a potential to be a novel treatment option to reduce arrhythmogenesis.

## 1. Introduction

Myocardial ischemia-reperfusion injury is associated with sympathetic hyperactivity in both experimental animal models (Hausenloy et al., 2012; van Waes et al., 2013) and in humans (Saffitz, 2008). Sympathoexcitation triggers acute cardiac electrophysiological changes that lead to malignant ventricular arrhythmias and sudden cardiac death (Schwartz et al., 1984). Modulation of the cardiac autonomic nervous system may be a potential treatment strategy against myocardial ischemia-induced sympathoexcitation.

Spinal cord neuromodulation therapies have recently emerged as therapeutic approaches for ventricular arrhythmias in pre-clinical and clinical research (Do et al., 2017; Howard-Quijano et al., 2017). Cardio-spinal autonomic neural reflexes are initiated by primary afferent neurons projecting to the dorsal horn of the thoracic spinal cord *via* dorsal root ganglion (DRG) (Foreman, 1999; Ardell et al., 2016). As such, DRG could serve as another anatomically accessible target for bioelectronic neuromodulation (Krames, 2015). Clinical trials have demonstrated the efficacy and effectiveness of DRG stimulation (DRGS) as a novel approach for relieving pain both clinically and pre-clinically (Deer et al., 2017; Koetsier et al., 2020). Recently it has been shown that DRGS decreased sympathetic outflow and reduced blood pressure (Sverrisdottir et al., 2020), but it has not been elucidated whether DRGS could reduce cardiac sympathetic excitation induced by acute myocardial ischemia and reperfusion injury.

We hypothesized that neuromodulation by DRGS would reduce cardiac sympathoexcitation and ventricular excitability induced by acute myocardial ischemia. Therefore, the primary aim of this study was to investigate the effect of DRGS on cardiac electrophysiology and ventricular arrhythmias triggered during acute myocardial ischemia. Our secondary aim was to study the cellular pathways in the spinal cord and DRG that are associated with DRGS during myocardial ischemia. Specifically, we examined if DRGS reduces ischemia-induced afferent neuronal input and neuronal activation in the thoracic spinal cord and apoptosis in the DRG, as cellular changes associated with reduction of cardiac sympathoexcitation and ventricular arrhythmias.

## 2. Materials and methods

The study protocol was approved by Institutional Animal Care and Use Committee (IACUC) and all experiments were conducted in compliance with the National Institution of Health Guide for the Care and Use of Laboratory Animals.

### 2.1. Animal preparations

Yorkshire pigs (*n* = 23, 11 males and 12 females), weighing 45 ± 1 kg, were included in the study. Animal preparations were conducted as previously reported (Howard-Quijano et al., 2017). Animals were sedated with Telazol (4 mg/kg, intramuscular) and Xylazine (2 mg/kg, intramuscular), underwent tracheal intubation, and were mechanically ventilated and general anesthesia was maintained with inhaled isoflurane (1 to 3%) during surgical preparation. A Prucka CardioLab recording system (GE Healthcare, Fairfield, CT, USA) was used for Heart rate (HR) and surface electrocardiogram (ECG) monitoring. The carotid and femoral arteries were catheterized for blood pressure monitoring and Millar catheter insertion (SPR-350). In addition, jugular and femoral veins were cannulated for saline infusion (10 ml/kg/hr) and drug administration. Arterial blood gas was checked every hour with adjustment of ventilation if needed to maintain acid-base equilibrium. Body temperature was maintained by external warmers. Dorsal spinal laminectomy was done in prone position to expose the spinal cord and left dorsal root of T2, median sternotomy was then performed supine to expose the heart, and for terminal experiment animals were in left lateral position for DRGS and cardiac ischemia induction. After the completion of surgical preparation, general anesthesia was transitioned to intravenous alpha-chloralose (50 mg/kg initial bolus followed by a 20 mg/kg/hr continuous infusion) for less impact on cardiac sympathetic excitability as we previously described (Dale et al., 2020; Omura et al., 2021). Depth of anesthesia was assessed throughout the experiments by monitoring corneal reflexes, jaw tone, and hemodynamic indices. At experiment end, animals were euthanized by inducing ventricular fibrillation *via* injection of potassium chloride under deep anesthesia.

### 2.2. Acute myocardial ischemia

Myocardial ischemia was performed as previously described (Howard-Quijano et al., 2017). A prolene suture was placed around the left anterior descending coronary artery (LAD) below the second diagonal branch of the LAD. The suture was ligated with a short polyethylene tubing segment to create myocardial ischemia for 1 h. Ischemia was confirmed by the presence of ST-segment elevations.

### 2.3. Dorsal root ganglion stimulation (DRGS)

**FIGURE 1:**
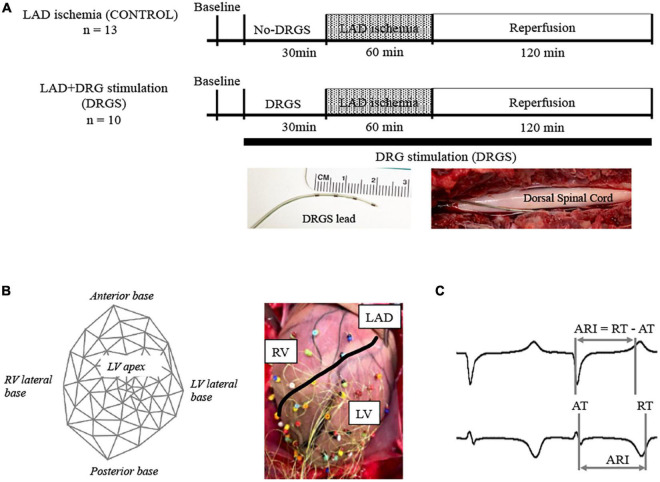
Experimental design. **(A)** Timeline of experimental protocol; Twenty-three Yorkshire pigs were randomized to two groups with or without dorsal root ganglion stimulation (DRGS). Single DRGstim catheter was placed at the left T2 DRG and the DRG was stimulated at 1 kHz of frequency. Acute myocardial ischemia was induced by ligating left anterior descending artery (LAD) for 1 h, followed by 2 h of reperfusion. DRGS was initiated 30-min before ischemia and continued throughout the experiment. Photographs of DRGS lead electrodes and representative placement of DRGS lead. DRGS lead was placed along with the spinal dorsal root. CONTROL: LAD ischemia (*n* = 13). DRGS: LAD ischemia + dorsal root ganglion stimulation (*n* = 10). **(B)** 56-electrodes heart mapping and representative cardiac electrogram; Polar map of 56-electrode heart mapping placed around the heart for measuring ventricular electrograms on the left and representative image on the right. **(C)** Representative cardio electrogram; ARI was measured as the difference between repolarization time (RT) and activation time (AT).

A 4-contact Axium™ DRG lead (St. Jude Medical, Minneapolis, MN, USA) was placed in the epidural space of the left DRG along the root of the second thoracic spinal level (T2), without removing the bone that covers the DRG. The stimulating electrode was enclosed in the body of intervertebral canal and ligaments which creates stabilization for the DRG lead (Figure 1A), similar to the clinical settings (van Velsen et al., 2018; Esposito et al., 2019). This stimulating lead was placed only in the DRGS group, with no placement in the CONTROL group. The T2 level was selected for DRGS as multiple cardiac afferent nerve fibers travel to the thoracic spinal cords through the C6-T6 DRGs bilaterally, with the largest numbers to the T2–T4 (Hopkins and Armour, 1989), and previous work has shown that cardiac afferents, as well as efferent output, are distributed symmetrically between the left and right sides (Yamakawa et al., 2016; Huang et al., 2017; Akgul Caglar et al., 2021) and thoracic DRGS at one level still gave cardioprotection (Kuwabara et al., 2021).

An A-M systems stimulator (Model 2100, A-M Systems, Sequim, WA, USA) was used to stimulate DRG. Motor threshold (MT) was assessed by increasing stimulus current intensity until muscle contractions were observed in the left shoulder, using a frequency of 2 Hz and pulse width of 0.4 ms. The average MT was 0.24 ± 0.03 mA in this study. Left T2 DRG was stimulated at frequency of 1 kHz, 0.03 ms pulse width, and current amplitude of 90% of MT.

### 2.4. Experimental protocols

The timeline of the experimental protocol is shown in Figure 1A. Twenty-three animals were randomized to two groups; LAD ischemia (CONTROL, *n* = 13) and LAD + dorsal root ganglion stimulation (DRGS, *n* = 10). After finishing the surgical preparation, the animal was positioned in the left lateral decubitus and a DRGS catheter was placed. Animals in both the CONTROL and DRGS groups were subjected to myocardial ischemia, but only the DRGS group received catheter-based neuromodulation.

### 2.5. Cardiac electrophysiology mapping and analysis

A 56-electrode mesh sock electrode system was placed around the heart and unipolar electrograms (0.05–500 Hz) were measured using a Prucka CardioLab electrophysiology mapping system (GE Healthcare, Fairfield, CT, USA) (Figure 1B) (Kuwabara et al., 2021). All physiological measures were recorded at- (1) baseline (BL), (2) during 30-min of DRGS and for 30-min in the control (No-DRGS group), and (3) during 1-h of acute myocardial ischemia (or until releasing the ligation). We assessed activation recovery interval (ARI), which has been shown as a surrogate of local action potential duration (Millar et al., 1985; Vaseghi et al., 2012). ARIs were calculated with customized software (iScalDyn, University of Utah, Salt Lake City, UT, USA) (Figure 1C), as previously described (Howard-Quijano et al., 2017). A 2-dimensional polar map was created with ARI data from the sock electrodes by using publicly available software (Map3d; Scientific Computing and Imaging Institute, University of Utah, Salt Lake City, UT, USA). Sympathetic stimulation is associated with shortened ARI duration and increased dispersion of repolarization (DOR) (Yamakawa et al., 2016). Raw ARIs analyzed by the mapping system are affected by HR. In this study, ARIs were corrected by HR using Bazett’s formula (Dahlberg et al., 2021). Ischemic regions were defined as the electrodes where ST elevations were seen. To ensure accuracy of ARI measurement, each electrogram with ST segment changes was both measured by semiautomated accepted software and then checked by hand following the guidelines described by Haws and Lux for ARI measurement in ischemia (Haws and Lux, 1990). Global epicardial DOR was analyzed using the variance of all mean ARIs. Epicardial dispersion is associated with a heterogeneity of repolarization time and increased risk for ventricular arrhythmias (Vaseghi et al., 2012).

To compare the electrophysiological changes during ischemia, the time point before the ischemia induction (30 min of No-DRGS or DRGS) was compared.

### 2.6. Tpeak-tend interval

Tp-Te interval was analyzed from the clearest limb lead in each animal. Tp-Te interval is an important marker for arrhythmogenicity in acute myocardial ischemia (Ozbek and Sokmen, 2019) and is also an independent risk predictor for sudden cardiac death (Yagishita et al., 2015). Tp-Te interval was assessed in limb leads with the clearest T wave at 200 mm/s paper speed as the average of five consecutive beats. Tp-Te interval was measured from the maximal T wave to the end of the T wave. The peak of the T wave was visually determined, and the end of the T wave was defined as the intersection of the tangent to the slope of the T wave and the isoelectric line (Figure 3A). Tp-Te interval was compared right before ischemia and at 30 min after ischemia onset to evaluate the arrhythmogenesis between the groups.

**FIGURE 2:**
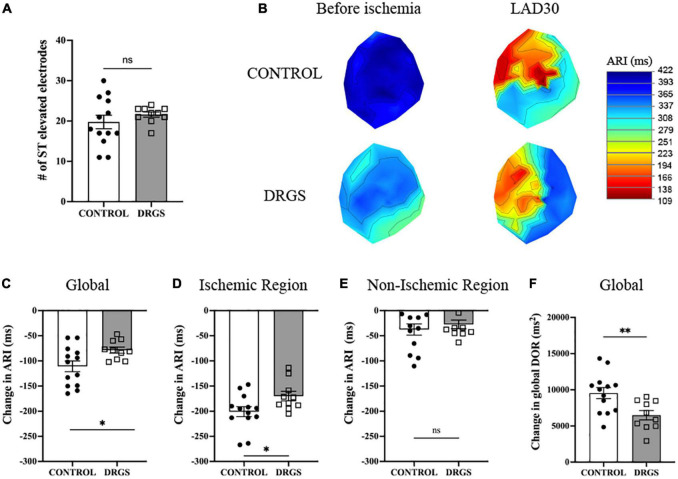
Change in electrophysiological parameters at 30 minutes of ischemia - **(A)** Numbers of ST elevation leads were comparable between the groups (*p* = 0.367). **(B)** Representative polar global activation recovery interval maps comparing before and after myocardial ischemia between the groups. **(C)** At 30 min after ischemia onset, change in activation recovery interval (ARI) was reduced by DRGS. DRGS attenuated cardiac sympathetic excitation in global heart (*p = 0.050). **(D)** Especially, in the ischemic region it was reduced by DRGS (*p = 0.020). **(E)** Change in ARI in the remote region of the heart from the ischemia was not reduced by DRGS (*p* = 0.741). **(F)** Change in global DOR was reduced by DRGS (*p = 0.016). DOR: dispersion of ARI. LAD: LAD ischemia (*n* = 11). DRGS: LAD ischemia + dorsal root ganglion stimulation (*n* = 9). Data are presented as mean ± SEM.

**FIGURE 3:**
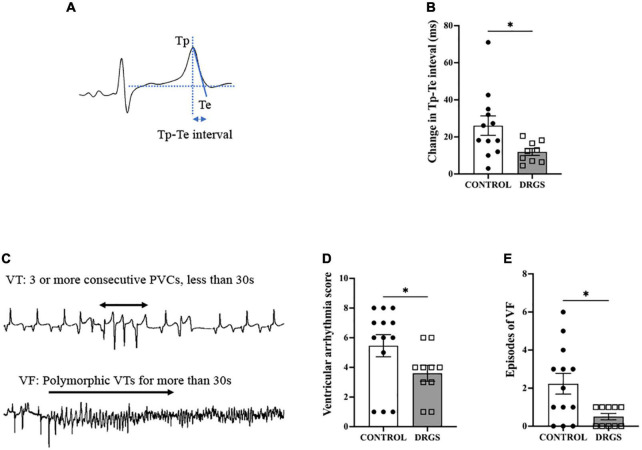
Tp-Te interval and Ventricular Arrhythmia assessment. **(A)** Methods of evaluating Tp-Te interval in the surface ECG based on the tangent method (Left). **(B)** DRGS reduced change in Tp-Te interval with significant difference (*p = 0.0407). **(C)** Representative tracings showing ventricular tachycardia (VT) and ventricular fibrillation (VF). **(D)** DRGS reduced ventricular arrhythmia score which demonstrates that DRGS had physiological effect on reducing cardiac arrhythmias during ischemia (*p = 0.038). **(E)** Episodes of ventricular fibrillation (VF) were reduced by DRGS during ischemia (*p = 0.024). CONTROL: LAD ischemia (*n* = 13). DRGS: LAD ischemia + dorsal root ganglion stimulation (*n* = 10). Data are presented as mean ± SEM.

### 2.7. ECG-based ventricular arrhythmia scoring system

Ventricular arrhythmias, which include premature ventricular contractions (PVCs), ventricular tachycardia (VT), and ventricular fibrillation (VF) episodes, were counted using Prucka CardioLab system. VT was classified as three or more consecutive PVCs and terminating spontaneously in less than 30 s (Figure 3C; European Heart Rhythm Association et al., 2006). A ventricular arrhythmia score (VAS) was calculated for each animal during myocardial ischemia according to the criteria previously reported (Curtis and Walker, 1988; Howard-Quijano et al., 2021). The scoring system assigns a numeric value based upon the severity of arrhythmia with larger scores demonstrating greater physiological severity. In brief, the VAS was characterized as: “0: No PVCs, VT, or VF,” “1: PVCs,” “2: 1–5 episodes of VT,” “3: > 5 episodes of VT or 1 episode of VF,” “4: 2–5 episodes of VF,” “5: > 5 episodes of VF.”

### 2.8. Hemodynamic assessment and surface ECG recordings

Hemodynamic measures were recorded using a five French Millar Mikro-Tip pressure transducer catheter (SPR-350) inserted into the left ventricle *via* the left carotid artery with continuous data recorded in an MPVS Ultra Pressure-Volume Loop System (Millar Instruments, Houston, TX, USA) throughout the experiment. Left ventricular systolic function was evaluated by the maximum rate of pressure change (dP/dt max). ECG was continuously recorded on Prucka CardioLab system (GE Healthcare, Fairfield, CT, USA).

### 2.9. Heart staining and measurement of area at risk and infarct size

Evans Blue and triphenyl tetrazolium chloride (TTC) were used for the heart staining as previously shown (Howard-Quijano et al., 2021). At the very end of the experiment after the animal was sacrificed, the LAD ligation was tied again and a cross clamp was placed on the aorta just above the base of the heart with the animal in the supine position. We made sure that the aorta was completely sealed in order to not leak the dye. Evans Blue dye was injected *via* the needle punctured right below the cross clamp with the heart being hand-massaged, then the heart was excised and sliced. The heart slices were then socked with TTC solution for 30 min. Area at risk (AAR) was defined as the area where it was not stained by Evans Blue, and it was measured as the ischemic insult in each animal. Infarct area was defined as the area where it turned to white color.

### 2.10. Immunohistochemistry

After myocardial ischemia and reperfusion (Figure 1A), T2 thoracic dorsal root ganglions and spinal cord were harvested for further analyses. Tissue preparation was performed as previously described (Howard-Quijano et al., 2021). Sections were processed in 4% paraformaldehyde (PFA, Thermo Fisher Scientific, Rockford, IL, USA) at 4°C for 2 days, followed by a 30% buffered (PBS) sucrose solution containing ∼ 0.01% sodium azide until tissues sank (11–14 days). The tissues were embedded with O.C.T. compound (Fisher Scientific, Fair Lawn, NJ, USA) and stored at −80°C until sectioning.

#### 2.10.1. Spinal cord assessment

Frozen tissues were cut at 35 μm thickness using a cryostat (CryoStar NX50; Thermo Fisher Scientific, Rockford, IL, USA). The slices were transferred to a six-well plate with six well inserts (Netwell Inserts, Corning, USA) and rinsed three times (5 min per time) in phosphate-buffered saline (PBS, PH 7.4; # BP24384, Fisher Scientific, Fair Lawn, NJ, USA) before being transferred to a blocking buffer for 1-h (PBS with 0.3% Triton x-100, 5% normal donkey serum or normal goat serum) at room temperature. Then they were incubated in PBS 0.3% Triton x-100 solution with primary antibodies (cFos; Abcam, #Ab190289, 1:6000 and NeuN; Millipore, #AbN90P, 1:1000) overnight at 4°C, followed by three-time wash (5 min per time) in PBS before being transferred to a PBS solution with secondary antibodies (Donkey anti-guinea Pig; Millipore, #AP193C, 1:500 and Donkey anti-rabbit; Invitrogen, #A21206, 1:2000) for 1-h at room temperature in the dark. Tissues were rinsed in PBS again (four times, 5 min per time) before being mounted and coverslipped with DAPI mounting medium (H-1500, Vector Laboratories, Burlingame, CA, USA). Double-label immunofluorescence was performed with Fos as a marker of cellular activation and NeuN as a neuron-specific marker. For double-labeling, slices were incubated in a solution containing both primary antibodies (Fos/NeuN). Negative controls with no primary antibodies were performed in all experiments to ensure antibody specificity.

#### 2.10.2. Dorsal root ganglion assessment

Frozen tissues were cut at 10 μm thickness using a cryostat (CryoStar NX50; Thermo Fisher Scientific, Rockford, IL, USA). The slices were put to a slide and rinsed three times (10 min per time) in phosphate-buffered saline (PBS, PH 7.4; # BP24384, Fisher Scientfic, Fair Lawn, NJ, USA) before being transferred to a blocking buffer for one and a half hours (PBS with 0.3% Triton x-100, 5% normal donkey serum) at room temperature. Then they were incubated in PBS 0.3% Triton x-100 solution with primary antibodies (CC3; Cell Signaling, #9661S, 1:800 and NeuN; Millipore, #AbN90P, 1:400) for 22–24 h at 4°C, followed by three-time wash (5 min per time) in PBS before being transferred to a PBS solution with secondary antibodies (Donkey anti-guinea Pig; Millipore, #AP193C, 1:500 and Donkey anti-rabbit; Invitrogen, #A21206, 1:2000) for 1-h at room temperature in the dark. Tissues were rinsed in PBS again (four times, 5 min per time) before coverslipped with DAPI mounting medium (H-1500, Vector Laboratories, Burlingame, CA, USA). Double label immunofluorescence was performed with cleaved caspase-3 (CC3) as a marker of apoptosis and NeuN as a neuron-specific marker.

#### 2.10.3. Image analysis

Sections were observed with a Nikon Eclipse Ti2 Inverted Microscope Systems using NIS-Elements AR Imaging Software V 5.10.01 (Nikon Instruments Inc., Melville, NY, USA). Microphotographs were taken using the 20× objective. All exposure times and processing procedures were identical across sample and treatment groups. Analysis was performed in blinded manner and protocols were standardized to avoid any potential experimental bias. For all IHC analyses, three slices were analyzed per spinal cord segment per animal. In spinal cord, cells that were NeuN +, cFos + and NeuN + /cFos + with DAPI were counted. The number of immunoreactive cells based on a uniformly set threshold across groups was counted within the superficial laminae (I-II) of the dorsal horn. For DRG assessment, manual cell quantification was completed using the Cell Counter plugin for ImageJ. Cells that were NeuN +, CC3 +, and NeuN + /CC3 + and compared between the CONTROL and DRGS groups.

### 2.11. Statistical analysis

All data are reported as mean and standard error (SE) for continuous data with normal distribution. Statistical normality was confirmed with the Shapiro-Wilk test. Differences in cardiac electrophysiologic measures between the CONTROL and DRGS groups were compared using unpaired *t*-test or Mann-Whitney tests per data normality. Two-way repeated-measures ANOVA with *post hoc* Holm-Sidak’s test was used to compare hemodynamic and electrophysiologic measures at different time points within the group and between groups. An unpaired *t*-test was performed to compare episodes of PVCs and area at risk, and a Mann-Whitney test was performed to compare arrhythmia score, episodes of VT or VF, and infarct size between the groups. Immunoreactive cell counts were compared using an unpaired *t*-test between the groups. Statistical analysis was done using Prism software (version 8, GraphPad Software Inc., San Diego, CA, USA). A two-tailed *p* value less than or equal to 0.05 (*p* ≤ 0.05) was considered to be statistically significant. The sample size was based on previous experience with a similar experimental model and design (Howard-Quijano et al., 2017, 2021).

## 3. Results

### 3.1. Effect of DRGS or time control before ischemia on cardiac electrophysiology

Thirty min DRGS alone did not change cardiac electrophysiological measurements. ARI and DOR also had no significant differences, as compared to baseline, within each group and between the groups (Table 1).

**TABLE 1:** Cardiac electrophysiological measurements.

	CONTROL (*n* = 13)			DRGS (*n* = 10)		
	BL	No-DRGS	LAD	BL	DRGS	LAD
Global ARI, ms	456 ± 14	469 ± 14	362 ± 13^†^	426 ± 15	425 ± 14	347 ± 11^†^
ARI in ischemic region, ms	460 ± 13	476 ± 14	259 ± 14^†^	434 ± 15	433 ± 14	264 ± 7^†^
ARI in non-ischemic region, ms	453 ± 14	463 ± 14	415 ± 10^†^	419 ± 17	420 ± 16	392 ± 13^†^
Global DOR, ms^2^	799 ± 122	739 ± 126	10285 ± 703^†^	419 ± 90	350 ± 69	6841 ± 592^†^,^‡^

No significant differences were seen in electrophysiologic parameters from baseline to DRG stimulation or time control. After ischemia ARI decreased and dispersion increased in both groups.

Between groups, global DOR was higher in LAD alone as compared to DRGS group.

^†^ = significant difference within group *p* < 0.0269, ^‡^ = significant difference between LAD and DRGS groups *p* < 0.0001.

All other p > 0.05. *n*; number of animals. Data are presented as mean ± SEM. BL; baseline. No-DRGS; 30 min time control without DRG stimulation. DRGS; 30 min DRG stimulation. LAD; 30 min acute ischemia. ARI, activation recovery interval; DOR, dispersion of repolarization.

### 3.2. Effect of DRGS during ischemia on cardiac electrophysiology

Two animals in the CONTROL group and one animal in the DRGS group had VF before 30 min of ischemia, therefore the data at 30 min of ischemia was analyzed and reported at the time right before VF happened for those animals.

The ischemic insult as measured by area at risk was greater in the DRGS group than in the CONTROL group (CONTROL: 24 ± 3.4%/left ventricle, DRGS: 31 ± 2.2%/left ventricle, *p* = 0.017), and the infarct size within the area at rick was comparable between the groups (CONTROL: 13 ± 3.5%, DRGS: 27 ± 7.0%, *p* = 0.0729).

In each group, as expected in ischemic model, ARI decreased, and DOR increased after ischemia (Table 1). There was no significant difference in the number of ST elevated electrodes during ischemia (Figure 2A). However, as shown in Figure 2B, the ischemic region had lower ARI in the CONTROL group, and when comparing the difference before and after ischemia, there was a significant reduction in the magnitude of global ARI shortening in the DRGS group (CONTROL, −111 ± 10.8 m vs. DRGS, −78.5 ± 6.0 ms, *p* = 0.0259, Figure 2C). Cardiac electrophysiologic changes were also examined regionally in the ischemic vs. non-ischemic region of the myocardium. In the ischemic zone, the change in ARI was significantly reduced by DRGS (CONTROL, −201 ± 9.8 ms, DRGS, −170 ± 9.4 ms, *p* = 0.0373, Figure 2D). On the other hand, there was no significant difference in the change in ARI in the non-ischemic region (CONTROL, −38 ± 11 ms. DRGS, −27 ± 8.6 ms. *p* = 0.503, Figure 2E). In addition, change in DOR during ischemia was also reduced by DRGS (CONTROL, 9546 ± 763 ms^2^, DRGS, 6491 ± 636 ms^2^, *p* = 0.0076, Figure 2F).

### 3.3. Effect of DRGS on arrhythmogenesis

#### 3.3.1. Tp-Te interval

During LAD ischemia, both groups increased Tp-Te interval (CONTROL: pre-ischemia, 34.3 ± 1.4 vs. post-ischemia: 59.7 ± 4.5 ms, *p* < 0.0001. DRGS: pre-ischemia, 34.5 ± 1.6 vs. post-ischemia: 46.7 ± 2.2, *p* = 0.014). However, in the between-groups comparison, DRGS significantly lowered the magnitude of change in Tp-Te interval during ischemia (Figure 3B).

#### 3.3.2. Ventricular arrhythmias

In total, 10 out of 13 animals in the CONTROL group and 5 out of 10 animals in the DRGS group had VF after LAD ischemia. One animal in the CONTROL group had three non-sustained VF episodes. The VAS and number of episodes of VF during ischemia were significantly reduced with DRGS as compared to acute myocardial ischemia alone (Figures 3D, E).

### 3.4. Hemodynamics

No significant differences were found in HR, SBP, LVESP, and dP/dt max, within the group and between the groups before ischemia (Table 2). During ischemia, there were no significant differences in hemodynamic measurements between the groups. Within-group, SBP and LVESP decreased more in the CONTROL group than the DRGS group after ischemia, while the other parameters had no significant changes (Table 2).

**TABLE 2:** Hemodynamic measurements.

	CONTROL (*n* = 13)			DRGS (*n* = 10)		
	BL	No-DRGS	LAD	BL	DRGS	LAD
HR, beats/min	79 ± 4	78 ± 4	84 ± 5	77 ± 4	81 ± 5	85 ± 3
SBP, mmHg	131 ± 6	131 ± 6	120 ± 7^†^	148 ± 6	149 ± 3	141 ± 3^†^,^‡^
LVESP, mmHg	106 ± 6	108 ± 5	102 ± 4	120 ± 9	120 ± 5	116 ± 2^‡^
dP/dt max, mmHg/s	2536 ± 280	2624 ± 281	2394 ± 327	2082 ± 291	2111 ± 217	2054 ± 199

No significant differences were seen in hemodynamic parameters from baseline to DRG stimulation or time control. SBP significantly decreased in both groups after ischemia. SBP and LVESP were lower after ischemia in the CONTROL than in the DRGS group.

^†^ = significant difference within group, *p* < 0.032, ^‡^ = significant difference between CONTROL and DRGS groups, *p* < 0.04.

All other *p* > 0.05. *n*; number of animals. Data are presented as mean ± SEM. BL, baseline. No-DRGS; 30 min time control without DRG stimulation. DRGS; 30 min DRG stimulation. LAD; 30 min acute ischemia. HR, heart rate; SBP, systolic blood pressure; LVESP, left ventricle end-systolic pressure; dP/dt max, maximum rate rise of LV pressure.

### 3.5. Immunohistochemistry

**FIGURE 4:**
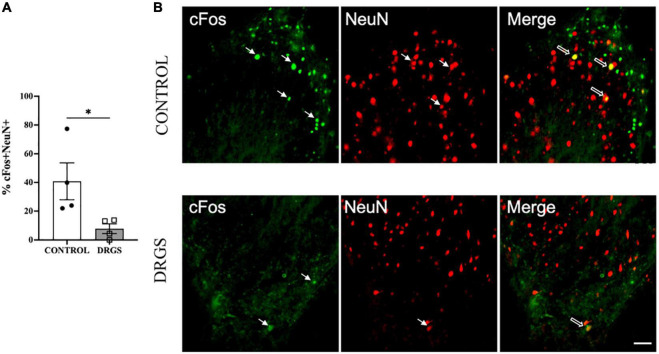
Double labeling of cFos with NeuN expressions in the T2 spinal cord. **(A)** DRGS decreased double positive cells with cFos and NeuN induced by myocardial ischemia (*p = 0.0476). **(B)** The representative images from both CONTROL and DRGS groups illustrate NeuN double-labeled cells with cFos. Small arrows indicate single-antibody marked cells, whereas big arrows indicate NeuN co-labeled with CC3 in the “merged” panels. CONTROL: LAD ischemia (*n* = 4). DRGS: LAD ischemia + dorsal root ganglion stimulation (*n* = 4). Data are presented as mean ± SEM. Image magnification, 20x and Scale bar, 50 μm.

In the superficial dorsal horn of the T2 spinal cord, myocardial ischemia-induced neural cell activation, represented by percent of cFos^+^/NeuN^+^ double-labeled cells (41 ± 13%), was significantly reduced by DRGS (7.8 ± 3.4%, *p* = 0.0476, Figure 4A, representative expressions shown in Figure 4B). In contrast, in the deep dorsal horn of the spinal cord (laminae lll - V, Vll, X), there was no significant difference in myocardial ischemia-induced neural cell activation between the groups (CONTROL: 46 ± 12%, DRGS: 65 ± 16%, *p* = 0.36).

**FIGURE 5:**
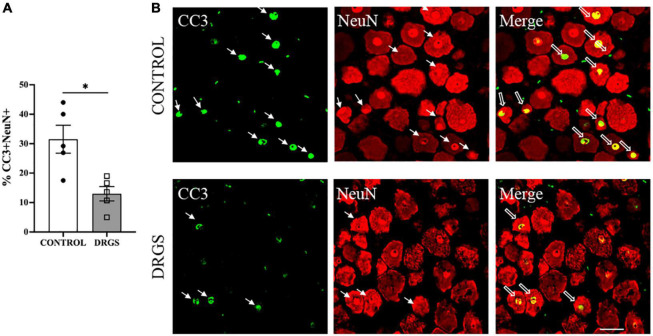
CC3 expressions in the left T2 DRG. **(A)** CC3 positive cells were significantly reduced with DRGS (A, *p = 0.0084). **(B)** The representative images from both CONTROL and DRGS groups illustrate NeuN double-labeled cells with CC3. Small arrows indicate single-antibody marked cells, whereas big arrows indicate NeuN co-labeled with CC3 in the “merged” panels. CONTROL: LAD ischemia (*n* = 5). DRGS: LAD ischemia + dorsal root ganglion stimulation (*n* = 5). Data are presented as mean ± SEM. Image magnification, 20× and Scale bar, 50 μm.

Myocardial ischemia induced neuronal apoptosis represented by percent of CC3^+^/NeuN^+^ double-labeled cells (34 ± 15%, 466 of 1,500 cells) in spinal cord dorsal horn, which was significantly reduced by DRGS (22 ± 10%, 255 of 1,641 cells, *p* = 0.0084, Figure 5A, representative expressions shown in Figure 5B).

## 4. Discussion

Despite advancements in medications or other techniques including implantable cardioverter-defibrillator (ICD), a significant number of patients with heart disease continue to experience arrhythmias and remain at risk for sudden cardiac death. There is a growing interest in utilizing neuromodulation of sympatho-vagal balance to reduce ventricular arrhythmias and sudden cardiac death (Franciosi et al., 2017). In a porcine model of acute myocardial ischemia, we found that thoracic DRGS attenuated myocardial sympathetic excitation and arrhythmogenesis. The major findings in this study suggest that DRGS at T2 level: (1) decreased ventricular sympathoexcitation with reduction in ischemia-induced ARI shortening, predominantly in ischemic myocardium, (2) reduced ventricular DOR and change in Tp-Te interval, (3) decreased ventricular arrhythmias with a reduction in the VAS and VF episodes during the ischemia, (4) decreased afferent neuronal activation in the superficial dorsal horn, and (5) decreased myocardial ischemia induced DRG cellular apoptosis. To our knowledge, this is the first report demonstrating the application of DRGS to modulate myocardial ischemia triggered sympathoexcitation and ventricular arrhythmias.

### 4.1. Effect of DRGS on cardiac sympathoexcitation and ventricular arrhythmias during myocardial ischemia

We found that DRGS attenuated cardiac ischemia induced sympathoexcitation and ARI shortening. There is a close correlation between action potential duration (APD) and ARI (Haws and Lux, 1990) and activation of myocardial sympathetic afferents during ischemia initiates activation of the sympathetic preganglionic neurons in the IML region in the thoracic spinal cord, which lead to increased sympathetic autonomic nervous system output and life-threatening arrhythmias (Fukuda et al., 2015; Franciosi et al., 2017).

Following myocardial ischemia, conduction blocks occur in the ischemic zone, which cause APD and *T*-wave alternans, leading to reentrant arrhythmias (Bernus et al., 2005; Downar et al., 2021). Also, after myocardial infarction, there is regional remodeling of the intrinsic cardiac nervous system, thus altering afferent neural signals and neural processing. These changes most frequently occur in the ischemic zones (border zones) and dynamically amplify the risk for ventricular arrhythmias (Vaseghi et al., 2012; Rajendran et al., 2016).

Cardiac primary afferent sympathetic neurons, with cell bodies located in DRG, are an essential component of this pathway for transmission of cardiac signals to the central nervous system during myocardial ischemia (Fukuda et al., 2015). Neuromodulation through bioelectronic DRGS can directly modulate dorsal horn neurons to decrease spinal neuronal sympathetic output to the heart (Sverrisdottir et al., 2020; Kuwabara et al., 2021). DRGS has also been previously shown to augment *T*-junction filtering through a reduction in action potentials from peripheral systems (Kent et al., 2018). Our results demonstrate that DRGS reduced ischemia induced cardiac sympathoexcitation likely through a reduction in the afferent neural transmission to the spinal cord.

In addition to reducing cardiac sympathoexcitation, this study also demonstrates that DRGS reduced ventricular arrhythmogenesis and resultant ventricular arrhythmias. Myocardial ischemia is accompanied by an increase in the dispersion of ventricular repolarization, primarily caused by disparate shortening of the action potential duration in areas most affected by ischemia (Behrens et al., 1997). Global DOR and Tp-Te interval are closely correlated and they have a strong link with arrhythmogenicity (Vaseghi et al., 2012, 2013), which is consistent with our data showing the reduced magnitude of change with DRGS. Previous results demonstrating a reduction in myocardial excitability created by extraventricular stimulus during DRGS is consistent with our current results (Kuwabara et al., 2021). Importantly, the VAS and episodes of VF during ischemia were significantly reduced by DRGS, which reinforces our electrophysiological data and the effects of DRGS on myocardial sympathoexcitation during ischemia.

### 4.2. Reduction of cFos in spinal cord and apoptosis in DRG with DRGS

The mechanism underlying reduction in ischemia-induced cardiac sympathoexcitation and arrhythmogenesis with DRGS in this study is postulated to be through modulation of myocardial afferent neural input into the dorsal horn of the spinal cord. This mechanism is supported by our data demonstrating a reduction in cFos + neuronal cell activation specifically in the superficial dorsal horn of the spinal cord. We and others have previously shown that cFos + neurons in the spinal cord are an accurate marker of neuronal activation during cardiac ischemia and neuromodulation with SCS (Howard-Quijano et al., 2021). Myocardial ischemia-sensitive ventricular sensory afferent neurons are capable of signaling to central neurons (Fukuda et al., 2015) and coronary artery occlusion has been shown to increase neuronal activity in the left T2 dorsal root ganglia with nerve endings projecting to the superficial dorsal horn of the spinal cord (Armour, 1999). Thus, the reduction in cFos + neurons in the superficial dorsal horn during ischemia with DRGS likely represents a reduction in ischemic afferent neural transmission to the spinal cord, which then leads to reduced efferent output from the thoracic IML neurons to the heart.

Myocardial ischemia activates the afferent neurons in the heart, and this excitatory response will trigger the spinal cord neural network and influence the intermediolateral (IML) neurons to cause the sympathoexcitation through the IML-sympathetic chain pathway (Dale et al., 2020; Omura et al., 2021). One of the potential mechanisms of action through which DRGS works is by blocking of the afferent signaling and preventing excitatory afferent signals from entering the spinal neural network during the ischemia, and thereby the sympathoexcitation is reduced or blunted. Cardiac neuraxis consists of three main levels of control, including intrinsic cardiac ganglia, intrathoracic extracardiac ganglia, and the central nervous system (Armour, 2004; Shivkumar et al., 2016). Brainstem, hypothalamus and the telencephalon areas of the brain are involved in the supraspinal neural processing of myocardial ischemia (Foreman, 1999; Janig, 2014). The sympathetic efferent pathway includes the neural hierarchy of supraspinal, cervical and thoracic spinal cord, sympathetic chain, and the intrinsic cardiac neural systems (Armour, 2004; Shivkumar et al., 2016). Spinal cord, intrathoracic extracardiac ganglia, and intrinsic cardiac nervous system have internal feedback loops which make them capable of receiving afferent information and providing the efferent outflow without communicating with the higher neural centers (Armour, 2004; Shivkumar et al., 2016). During the myocardial ischemia, the afferent neurons are activated (Salavatian et al., 2019) which will directly (through the internal reflex loop) and indirectly (from supraspinal to spinal cord to sympathetic chain and eventually to the intrinsic cardiac nervous system) trigger the sympathoexcitatory response. DRGS modulates the afferent pathway at the level of the spinal cord, and therefore, it has the potential to mitigate the sympathoexcitation that is modulated through the spinal cord.

Additionally, we also found a reduction in neuronal apoptosis in the DRG with DRGS, as demonstrated by a downregulation of neuronal CC3 expression. Previous reports have demonstrated that myocardial ischemia can lead to induction of inflammatory gene expression and/or neuronal apoptosis in different components of the cardio-spinal reflex pathway, including spinal cord, stellate ganglion, and DRG (Gao et al., 2017; Wang et al., 2018). Thus, these evidence and current results suggest that DRGS’s anti-arrhythmic effects, in part, may be due to its ability to mitigate cardiac ischemia-induced loss of neuron cell viability. However, further studies are needed to determine the precise mechanisms through which DRGS impacts DRG cells.

### 4.3. Clinical implications

Our study has demonstrated the cardiac protective effects of DRGS, however, the clinical translation is still premature and requires further investigations. We were able to show the effectiveness of DRGS in this study, where DRGS could modulate autonomic imbalances and reduce arrhythmogenesis in the ischemic myocardium in a healthy porcine model. Porcine models are very effective for cardiovascular research because they have similar physiology, heart size, immune system, anatomy, and coronary circulation also resembles human (Spannbauer et al., 2019).

While there are multiple modalities of neuromodulation available (Koetsier et al., 2020), we used 1 kHz high-frequency DRGS in this study based on its clinical efficacy and reduced side effect profile compared to SCS. High-frequency SCS is thought to be a more promising strategy as it has less paresthesias in its clinical application for pain therapies (North et al., 2016) and a recent clinical study showed high-frequency DRGS also worked well to relieve pain without paresthesia (Billet et al., 2017). Prior studies shows that there is no significant difference between low and high-frequency DRGS (Koetsier et al., 2020). It has also been shown that even the conventional DRGS causes less paresthesia compared to the SCS during the stimulation (Deer et al., 2019) and it also improves over time (Mekhail et al., 2020). This indicates that the use of high-frequency stimulation in the kilohertz range, which is usually associated with a higher power consumption, only for the purpose of avoiding paresthesia would not be necessary. In our previous report, we demonstrated that both 20 Hz and 1 kHz DRGS reduced the arrhythmogenicity induced by extra-ventricular stimuli with S1/S2 pacing with no superiority (Kuwabara et al., 2021). Additionally, there are currently no optimal stimulation parameters described for DRGS, especially during myocardial ischemia and more studies are needed to distinguish the therapeutic differences between conventional and kilohertz DRGS in more detail.

Interestingly, we were able to demonstrate efficacy of DRGS while stimulating DRG at a single thoracic level (T2) on the left side supporting results from previous reports that suggest both left or right cardio-spinal neural reflex pathways from the heart contribute to transmission of sympathetic afferent inputs to upper thoracic dorsal spinal columns. Recent evidence has shown that cardiac sensory soma is distributed symmetrically between the left and right sides of DRG (Akgul Caglar et al., 2021). Further, in porcine model, no change in the magnitude of ARI shortening were seen between the left or right spinal dorsal root transections from T1 to T4 spinal levels following sympathetic activation by stellate ganglion stimulation (Yamakawa et al., 2016). While we did not evaluate if there would be an incremental benefit of multiple spinal level or bilateral DRGS, the simplicity of our approach lends to better clinical translation.

### 4.4. Limitations

We showed the effectiveness of DRGS in a myocardial ischemia porcine model, but this study has some limitations. Anesthesia with isoflurane can suppress nerve activity and change cardiac electrophysiology. However, we discontinued isoflurane after finishing surgical procedures and used alpha-chloralose to minimize the autonomic effects. The protocol remained similar in both the groups, thus the results seen in this study should be least impacted. Also in this study, DRGS was initiated prior to ischemia and we showed DRGS decreased cardiac sympathoexcitation during ischemia, which we think would be considered as the potential strategy in a clinical situation where a second acute myocardial ischemia and/or recurrent ventricular arrhythmia happen. While we performed this study in a healthy porcine model with acute ischemia, there is a possibility that the result may not be directly applicable in the setting of chronic myocardial ischemia/infarction. In this study, the DRGS lead was not placed in the CONTROL group, but the surgical preparation including the laminectomy was performed similarly in both groups. We do not expect that the placement of the DRGS lead in the CONTROL group would significantly change the results of the study as the DRGS lead placement alone in the pain studies could not suppress the activity of sensory neurons to provide the therapeutic effect (Yu et al., 2020). Finally, our molecular findings provide important insight into how DRGS may be reducing ischemia-induced cardiac arrhythmias, further studies are needed to elucidate the specific molecular pathways through DRGS could work in the DRG or spinal cord.

## 5. Conclusion

In a porcine model, we have demonstrated the efficacy of DRGS to decrease myocardial ischemia-induced cardiac sympathoexcitation and reduction of ventricular arrhythmias through modulation of primary afferent signaling to the spinal cord, neuronal cell activation in the superficial dorsal horn, and a reduction in neuronal apoptosis in the DRG. This study provides insights for the further considerations of DRGS as a novel neuromodulation option and potential future translation to patients with severe ventricular arrhythmias triggered by myocardial ischemia.

## Data availability statement

The raw data supporting the conclusions of this article will be made available by the authors, without undue reservation.

## Ethics statement

The animal study was reviewed and approved by Institutional Animal Care and Use Committee (IACUC) at University of Pittsburgh.

## Author contributions

YK, KH-Q, and AM conceived and designed the research. YK and TY conducted the experiments. YK analyzed the data and prepared the figures. YK, KH-Q, SiS, SaS, and AM interpreted the results of experiments and drafted the manuscript. All authors reviewed and approved the final version of the manuscript.
